# Hunger

**Published:** 1890-06

**Authors:** 


### HUNGER.

This is a sensation which correctly indicates the real wants of the system, at least, if the stomach is in a normal condition. It was manifestly intended that this should fairly represent—as the thermometer does the temperature—to what extent the body has been wasted, its tissues actually destroyed by exercise, the need of physical and mental food being indicated by this "foodometer" for the repair of such waste. Hard work, violent efforts, mental labors included, increase the appetite, simply because such unusual labors waste the tissues unusually.

On the other hand if the usual amount of labor is diminished, there is naturally a diminished appetite, mercifully indicating a demand for less food. It is for this reason that those who have been very active in business life, generally live but a short time after leading an indolent life, particularly those who do little save to eat and sleep. They overpower the organs of digestion, practically starve themselves. The digestive organs, in their debilitated state, being unable to appropriate enough to meet the wants of the system.

On the principle of the formation of bad habits, by the use of intoxicants, tobacco, etc., the appetite may become so vitiated, so revolutionized, that what is regarded as hunger will not fairly represent the true wants of the system, and should never be taken as a guide in the matter of eating. Thus, when one habitually uses too much food, more than the system demands, gradually learning to eat more by one-third than usual, a habit is formed, an abnormal appetite created, the result of which is an artificial hunger, or what is called hunger, in no sense reliable. A similar result is produced when rich and unnatural food is taken, food which satisfies a false appetite, this sensation of supposed hunger being no more reliable than the tobacco user's desire for the "filthy weed." Unnatural longings are induced by these causes, often mistaken for hunger, the gratification of which necessarily leads to dyspepsia and various digestive disturbances.

